# Poly(2,5-bis(*N*-Methyl-*N*-Hexylamino)Phenylene Vinylene) (BAM-PPV) as Pretreatment Coating for Aerospace Applications: Laboratory and Field Studies

**DOI:** 10.3390/ma7128088

**Published:** 2014-12-17

**Authors:** Peter Zarras, Diane Buhrmaster, Cindy Webber, Nicole Anderson, John D. Stenger-Smith, Paul A. Goodman

**Affiliations:** 1 Naval Air Warfare Center Weapons Division (NAWCWD), Polymer Science & Engineering Branch (Code 4L4200D), 1900 N. Knox Road (Stop 6303), China Lake, CA 93555, USA; E-Mails: cynthia.webber@navy.mil (C.W.); nicole.anderson@navy.mil (N.A.); john.stenger-smith@navy.mil (J.D.S.-S.); paul.goodman@navy.mil (P.A.G.); 2 Air Force Research Laboratory/Logistics Systems Support Branch Coatings Technology Integration Office, UDRI Contractor Support, Bldg 1661, Rm C-110, Wright-Patterson AFB, University of Dayton Research Institute, Nonstructural Materials Division, 300 College Park, Dayton, OH 45469, USA; E-Mail: diane.buhrmaster.ctr@us.af.mil

**Keywords:** poly(2,5-bis(*N*-methyl-*N*-hexylamino)phenylene vinylene) (BAM-PPV), hexavalent chromium (CrVI), passivation, accelerated weathering tests, field studies

## Abstract

In this study, an electroactive polymer (EAP), poly(2,5-bis(*N*-methyl-*N*-hexylamino)phenylene vinylene) (BAM-PPV) was investigated as a potential alternative surface pretreatment for hexavalent chromium (Cr(VI))-based aerospace coatings. BAM-PPV was tested as a pretreatment coating on an aerospace aluminum alloy (AA2024-T3) substrate in combination with a non-Cr(VI) epoxy primer and a polyurethane Advanced Performance Coating (APC) topcoat. This testing was undertaken to determine BAM-PPV’s adhesion, corrosion-inhibition, compatibility and survivability in laboratory testing and during outdoor field-testing. BAM-PPV showed excellent adhesion and acceptable corrosion performance in laboratory testing. The BAM-PPV aerospace coating system (BAM-PPV, non-Cr(VI) epoxy primer and polyurethane APC topcoat) was field tested for one year on the rear hatch door of the United States Air Force C-5 cargo plane. After one year of field testing there was no evidence of delamination or corrosion of the BAM-PPV aerospace coating system.

## 1. Introduction

The Department of Defense (DOD) relies on multiple-component protective coating systems to maintain the operational readiness of military aircraft. Current high-performance aerospace coating systems (pretreatment, primer and topcoat) for DOD applications center on the use of hexavalent chromium (Cr(VI)) in both the pretreatment and primer layers. The pretreatment coating is a conversion coat that is Cr(VI)-based and the primer contains Cr(VI)-based inhibitors (e.g., strontium chromate (SrCrO_4_)). The pretreatment and primer are coated with a polyurethane topcoat to complete the aerospace coating. This DOD aerospace coating has been developed over many years to meet the strenuous challenges of corrosion protection, adhesion and weathering encountered by military platforms. DOD aerospace platforms such as the F-18, F-16, F-22, Joint Strike Fighter, MV-22, CV-22, H-60, C-141, C-130, C-5 and P-3 Orion aircraft use these coatings for corrosion protection and to ensure mission readiness.

Chromate conversion coatings (CCCs) and Cr(VI) primers are effective at inhibiting corrosion, because they are capable of “self-healing”. The soluble Cr(VI) present in CCCs and to a lesser degree in Cr(VI) primers acts as a reservoir for mobile Cr(VI). These mobile Cr(VI) species are capable of migrating to defects and inhibiting further corrosion by creating a passivating layer at the defect site [1,2,3,4,5,6]. Cr(VI) compounds are known carcinogens and are toxic to both humans and the environment [7]. Chronic inhalation of Cr(VI) compounds increases the risk for lung cancer. Soluble species can cause or exacerbate contact dermatitis. Ingestion can cause irritation and ulcers of the stomach and intestine and Cr(VI) is transported into cells via the “sulfate transport” mechanism [8]. Due to its toxicity and persistence in the environment, Cr(VI) is highly regulated. Therefore, alternative non-toxic pretreatment coatings are under development to replace Cr(VI) with a more benign material.

Recent work on alternatives to Cr(VI) coatings have produced surface pretreatments or conversion coats such as Trivalent Chromium Pretreatment (TCP), Prekote and Alodine 5200/5700^®^ [9,10,11,12,13]. These systems have been used as alternatives to CCCs with varying degrees of success. When they are combined with non-Cr(VI) epoxy primers, these alternatives have not performed as well in aerospace applications as coatings containing Cr(VI). Typically, these alternatives have been unable to meet the minimum standard of 2000 h of neutral salt spray (NSS) exposure before failing. The failure modes include corrosion, blistering or delamination of the coating.

Over the past 25 years, published evidence that EAPs especially, polyaniline (PANI) can inhibit corrosion has come from the pioneering work of Mengoli [14] and DeBerry [15]. Mengoli showed that EAP coatings such as PANI deposited onto iron anodes by electropolymerization of aniline resulted in an adherent and corrosion inhibiting film. Further work by DeBerry in 1985 showed that PANI films electrochemically deposited from an aniline monomer solution onto stainless steel provided corrosion protection from sulfuric acid environments. These works and others demonstrated that the PANI film provided anodic protection, thus maintaining a native passive film on the steel [15,16]. There are numerous laboratory studies in which EAPs deposited onto metals exhibit corrosion inhibition under a variety of corrosive environments [17,18,19]. Additionally, EAPs and chromium species undergo oxidation at similar potentials, which means that corrosive species that normally react with the chromium coatings will likely also react with EAPs [20].

**Figure 1:**
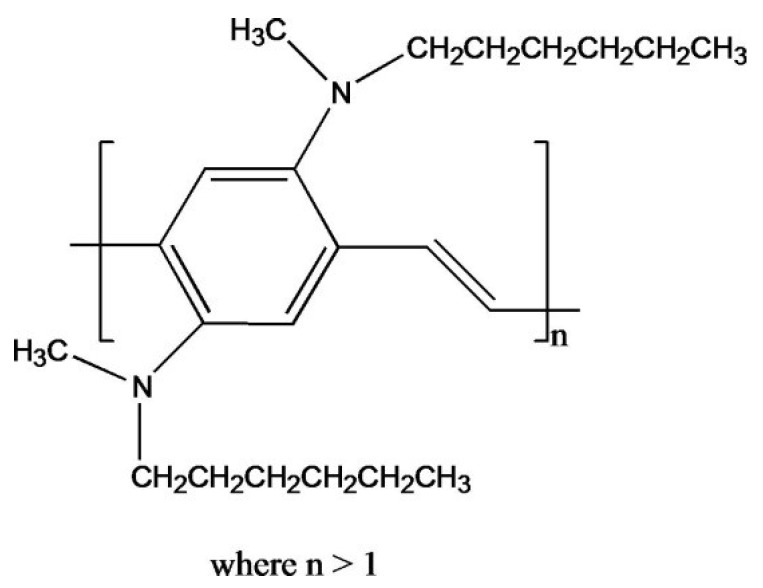
Structure of BAM-PPV.

Poly(2,5-bis(*N*-methyl-*N*-hexylamino)phenylene vinylene) (BAM-PPV), Figure 1, has been examined as a replacement for CCC’s on AA2024-T3 alloys. Neutral salt spray (NSS) exposure testing at the Naval Air Warfare Center Weapons Division (NAWCWD), China Lake, California has shown that BAM-PPV coatings deposited onto AA2024-T3 can prevent corrosion ≥336 h. A DOD CCC replacement requires a minimum of 336 h of NSS exposure testing without evidence of corrosion, blistering or delamination to be considered as an alternative [21,22,23].

Studies in which advanced electrochemical techniques were used to examine BAM-PPV’s corrosion protection mechanism have been published previously. Scanning vibrating electrode technique (SVET) and electrochemical noise method (ENM) measurements showed that BAM-PPV films may passivate the metal surface in Dilute Harrison Solution (DHS) [24].

Published studies using SVET experiments performed on BAM-PPV coatings on AA2024-T3 coupons showed that all current flow was forced to occur only within the defect area. The magnitude of the oxidation current increased with time, reaching 80 μA/cm^2^ after 6 h and 10 min of immersion in DHS. The corresponding optical micrograph with overlaid current density vectors, published in [24], indicated that the oxidation current was localized at the visually corroded area of the defect, while the reduction current appeared to occur both within the defect and to some extent at the polymer film surface. This observation may be due to the polymer undergoing oxidation/doping. Thus, rendering itself sufficiently conductive to mediate electron transfer from the metal/polymer interface to polymer/solution interface. The oxidation current then slowly decreased and reached 10 μA/cm^2^ after 24 h of immersion. The corresponding electronic or electrochemical interaction between the polymer and the metal provides evidence for passivation of the metal.

Thin film BAM-PPV coatings (film thickness 0.4 μm) on AA2024-T3 coupons were measured using ENM and the magnitudes of the measured noise resistances were quite low (1 × 10^4^–1 × 10^5^ Ω) [24]. Cross-scratched samples of BAM-PPV coated samples showed an initial value of about 5 × 10^4^ Ω noise resistance on the first three days of immersion in DHS. The resistance dropped to 1 × 10^4^ Ω by the tenth day of immersion and maintained this steady resistance through 42 days. For the non-scratched sample the average initial noise resistance (1 × 10^5^ Ω) was higher than that of cross-scratched samples. The fluctuating noise resistance as a function of immersion time may due to the formation/breakdown activities of a passive oxide film at the metal/BAM-PPV polymer interface. Some corrosion products were found after 7 days of immersion. By day 30 the samples had significant buildup of corrosion indicating complete coating failure.

Electrochemical impedance spectroscopy (EIS) measurements on the stability of BAM-PPV films at various pH’s (Tris, pH 8.1, and acetate, pH 4.5, buffer solutions) at room temperature showed that BAM-PPV was robust without evidence of delamination or blistering [22,23]. The BAM-PPV films also exhibited purely capacitive behavior with a slightly higher pore resistance (*R*_po_) than that observed for the bare AA2024-T3 substrate. In the case of the bare AA2024-T3 substrate, a thin oxide layer is present that strongly adheres to the metal surface and acts as a protective passivation layer. The higher pore resistances measured for the BAM-PPV coated AA2024-T3 substrate suggest that the polymer layer also contributes to the electrical properties at the aluminum/liquid junction. In effect, the polymer coating appears to function as a resistor in series with the oxide layer on the aluminum. The stability of this additional layer is indicated by the persistently higher *R*_po_ values obtained with the BAM-PPV coated panels *versus* the bare aluminum. This evidence suggests that the electronic properties of the BAM-PPV layer are not significantly altered by the buffer solution over the course of the experiments. Further EIS studies on the resistive nature of the BAM-PPV coating *versus* CCC on AA2024-T3 coupons showed that the impedances at low frequencies do not significantly change within the first six months of exposure to the 0.5 M NaCl solution. The total resistances do not deviate significant from 1 × 10^4^ to 1 × 10^5^ Ω regardless of the coating.

The previously published electrochemical results were encouraging and demonstrated that BAM-PPV warranted further investigation as a practical coating replacement for Cr(VI)-based systems. The performance of BAM-PPV aerospace coatings in adhesion, accelerated weathering and outdoor exposure tests are reported here. These results, in combination with the previously reported work, show that BAM-PPV coatings perform similarly to Cr(VI)-based systems in both laboratory and field scale testing, and because of their high performance, BAM-PPV coatings provide a good alternative to Cr(VI)-based coatings.

## 2. Results and Discussion

The following Table 1 summarizes the results from the laboratory and outdoor exposure testing for the BAM-PPV pretreatment and control. BAM-PPV coating was 2 μm in thickness, followed by 37.5 µm of non-Cr(VI) epoxy primer (MIL-PRF-23377, Type I, Class N) and 52.5 µm of a polyurethane topcoat (MIL-PRF-85285, Advanced Performance Coating (APC)). This coating was measured against a control that consisted of a CCC (<0.1 μm) followed by 37.5 µm of a Cr(VI) epoxy primer (MIL-PRF-23377, Type 1, Class C) and 52.5 µm of a polyurethane topcoat (APC), which is highlighted in green.

**Table 1:** Results from Laboratory Testing of BAM-PPV Coatings *vs.* Control.

Test method	Control coating system	BAM-PPV coating system
ASTM D 3359 [25] Crosshatch Adhesion	4B (Pass) (% area removed <5%)	4B (Pass) (% area removed <5%)
ASTM D 3359 [25] Wet-tape Adhesion	5A (pass)	5A (pass)
ASTM D 4541 [26] PATTI Adhesion	1759 psi (±100 psi)	1141 psi (±100 psi)
ASTM B 117 [27] NSS Exposure	Rating (1,0,0) (Pass)	Rating (1,2,0) (Low)
ASTM G 155 [28] Xenon Arc	Δ*E* = 0.6	Δ*E* = 0.3
ASTM D 1014 [29] Outdoor Exposure	12 months exposure, Rating = 0,0,0 (Pass)	12 months exposure, Rating = 0,0,0 (Pass)
	24 months exposure, Rating = 0,0,0 (Pass)	24 months exposure, Rating = 0,0,0 (Pass)

### 2.1. Adhesion Testing

BAM-PPV samples were analyzed for their adhesion quality both qualitatively and quantitatively. For all of the coating tests discussed below the BAM-PPV aerospace coatings consisted of AA2024-T3 substrates coated with 2 µm of BAM-PPV, primer and APC topcoat and all samples were measured against the Cr(VI) control coating.

#### 2.1.1. Crosshatch Adhesion Testing Results

The crosshatch adhesion test follows ASTM D 3359 [25] and provides a qualitative evaluation of a coating’s adhesion performance while dry through visual examination, and is performed prior to laboratory testing in an accelerated weathering chamber [25]. The description of the rating scale, as taken from ASTM D 3359 [25], is given in Table 2.

**Table 2:** ASTM D 3359 Method B, Crosshatch adhesion rating scale with description and percent (%) area removed.

Rating Scale	Description	Percent (%) Area Removed
5B	The edges of the square are completely smooth none of the squares of the lattice are detached	0%
4B	Small flakes of the coating are detached at intersections less than 5% of the area is affected	<5%
3B	Small flakes of the coating are detached along edges and at the intersections of cuts	5%–15%
2B	The coating has flaked along the edges and on parts of the squares	15%–35%
1B	The coating has flaked along the edges of the cuts in large ribbons and whole squares have detached	35%–65%
0B	Severe flaking and detachment across entire square	>65%

Both the BAM-PPV and the control coatings were scribed with a 2-mm spacing crosshatch blade. There were three replicates for each coating system. The control system received a pass with a rating of 4B, and BAM-PPV also passed this laboratory test with an adhesion rating of 4B, see Table 2. Both systems showed small flakes of their respective coating that were detached at the intersections of the scribed areas. The amount of coating that was removed from the sample, as determined by visual inspection, was <5%.

#### 2.1.2. Wet-Tape Adhesion Testing Results

The wet-tape adhesion test is another qualitative measurement of the adhesion performance for organic systems immersed in water for 24 h. The rating system from ASTM D 3359 [25], Method A, was used to rate the coatings as a pass/fail [25]. A fail rating would preclude any further analysis of the samples for military applications and thus the test is essential. The rating scale with descriptions is found in Table 3. The test was performed on three replicates of each coating system and both the control and BAM-PPV received a pass with a rating of 5A, see Table 1. The 5A rating indicated that there was no evidence, upon visual inspection, that any coating was removed from the substrate after immersion in water.

**Table 3:** ASTM D 3359 Adhesion, Method A, rating scale with description.

Rating Scale	Description
5A	no peeling or removal of coating
4A	trace peeling or removal along incisions or intersections
3A	jagged removal along incisions up to 1/16 inch on either side
2A	jagged removal along most of incisions up to 1/8 inch
1A	removal from most of the inscribed area
0A	removal beyond the inscribed area

#### 2.1.3. PATTI Adhesion Results

The Pneumatic Adhesion Tensile Test Instrument (PATTI) test is a quantitative test of the adhesive strength of a coating and the procedure is defined by ASTM D 4541 [26]. For this test, a tensile stress is applied to the coating until a physical failure occurs. The failure can be either adhesive (inter-layer) or cohesive (intra-layer). Six replicates each of the control and BAM-PPV systems were tested. The control coating required an average tensile stress of 1729 psi to cause failure, and the failure was observed to be cohesive in nature and within the epoxy primer layer. The AF has determined that the appropriate adhesion value for the standard CCC, Cr(VI) epoxy primer, and APC polyurethane topcoat system is between 1600 and 1800 psi.

The BAM-PPV coating system required an average tensile stress of 1141 psi to cause failure. The failure of the BAM-PPV coating was also cohesive, but the failure was within the pretreatment (BAM-PPV) layer.

The results of the PATTI testing showed that that BAM-PPV demonstrated acceptable adhesion strength when compared to the control, see Table 1. Each coating system failed by a cohesive mechanism which indicates that interlayer bonding is not an issue. The BAM-PPV showed lower adhesion values but this is not considered a failure, only that the BAM-PPV coatings showed lower adhesion strength when compared to the control. The failure within the pretreatment layer in the BAM-PPV sample could be problematic if the strength of the pretreatment layer changes over the lifetime of the coating. However, based on the results of the accelerated weather and field testing that are discussed below, it appears that the failure mode and lower adhesion strength for the BAM-PPV samples found in PATTI testing does not affect the practical performance of the coating.

### 2.2. Accelerated Weathering

#### 2.2.1. Neutral Salt Spray (NSS) Test Results

BAM-PPV and control samples were scribed and exposed to a NSS in accordance with ASTM B 117 [27]. The panels were examined visually for coating adhesion and evidence of corrosion after 2000 h NSS exposure. The samples were assigned ratings on a scale of 0–5 for three different observable phenomena: scribe appearance, undercutting and blistering [27]. The definitions for the 0–5 scales are given in Table 4. In short, a score of 0 essentially indicates a perfect performance and 5 indicates a complete failure. The ratings are derived from ASTM methods and the United States Air Force uses this ratings system as a comparative scale for corrosion inhibition materials. The ratings for the BAM-PPV samples were 1, 2, 0, representing scribe appearance, undercutting and blistering, respectively. For comparison, the control samples were rated 1, 0, 0. As per the testing standard, the rating of “2” in the undercutting category, which means the coating lifted near the scribed area by 3 mm or less, resulted in the BAM-PPV system receiving a “low” test result. The control samples showed no undercutting and therefore received an “acceptable” test result.

**Table 4:** Scribe, undercutting and blistering appearance rating system.

Rating Scale	Scribe Appearance	Undercutting Appearance	Blistering Appearance
0	Bright and clean	No lifting of coating	None
1	Staining no corrosion build-up	Lifting or loss of adhesion up to 1/16 inch (2 mm)	Very small
2	Minor corrosion build-up	Lifting or loss of adhesion up to 1/8 inch (3 mm)	Small
3	Moderate corrosion build-up	Lifting or loss of adhesion up to 1/4 inch (6 mm)	Medium
4	Major corrosion build-up	Lifting or loss of adhesion up to 1/2 inch (13 mm)	Large
5	Severe corrosion build-up	Lifting or loss of adhesion beyond 1/2 inch (>13 mm)	Delamination

**Figure 2:**
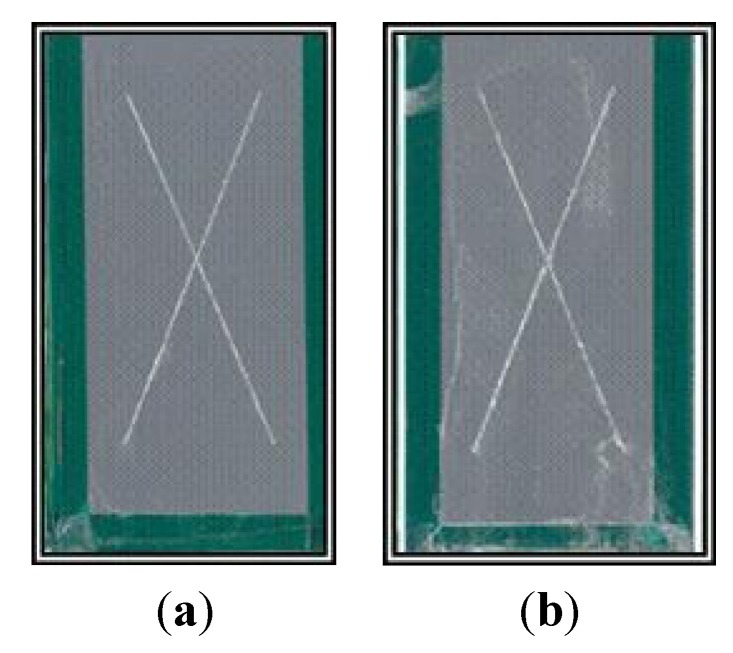
Photos after 2000 h of NSS exposure testing for (**a**) control; and (**b**) BAM-PPV coated AA2024-T3 coupons.

The test results show that BAM-PPV provides acceptable corrosion inhibition when compared to the control via visual inspection. Each sample showed staining within the scribed area, but no visible buildup of corrosion. Neither coating blistered after 2000 h of NSS exposure testing, see Table 1. The control and BAM-PPV samples were photographed after the 2000 h NSS exposure testing and the photographs are shown in Figure 2. Though the results of this test indicate that BAM-PPV performed equivalently to the Cr(VI)-based coatings for corrosion inhibition, the duration of the inhibition will not be equivalent. The CCC and primer in the control sample act as a reservoir for mobile Cr(VI), and the high concentration of mobile Cr(VI) allows the chromium based coatings to re-passivate defects in the surface [1,2,3,4]. BAM-PPV also passivates the AA surface, as shown in previous studies, but its duration is limited, due to the lower concentration of sacrificial redox reaction sites, and cannot match the long term corrosion-inhibiting properties of Cr(VI) [1,2,3,4,5,6,21,22,23,24].

#### 2.2.2. Xenon arc Accelerated Weathering Test Results 

Limiting the amount of color change a corrosion inhibition coating exhibits during its lifetime is an important aesthetic property, in fact the MIL-PRF-85285 requirement for the APC topcoat states that “topcoated systems [must] demonstrate a color change (Δ*E*) of no more than 1 unit after 500 h of exposure to xenon arc light” [28]. Xenon arc accelerated weathering tests allow one to measure the perceived color change of a sample, and were thus performed on both control and BAM-PPV coated samples. Color values are measured in CIELAB units, and the Δ*E* is calculated, per ASTM D 2244 [30], from the following equation:

(1) $$\Delta E=\sqrt{{\left(\Delta L\right)}^{2}+{\left(\Delta a\right)}^{2}+{\left(\Delta b\right)}^{2}}$$

where Δ*L* = *L*_final_ − *L*_initial_; *L* = the white to black area in color space and is located on the *z*-axis. It is always a positive value:

Δ*a* = *a*_final_ − *a*_initial_ (2)

where *a* = the green to red area in color space, located on the *y*-axis. It can be a negative value (more green) or a positive value (more red) in color space:

Δ*b = b*_final_*− b*_initial_ (3)

where *b* = the blue to yellow area in color space, located on the *x*-axis. It can be a negative value (more blue) or a positive value (more yellow) in color space.

Both the BAM-PPV and control received a pass rating in this test, with ΔE values of 0.3 and 0.6, respectively, see Table 1.

### 2.3. Natural Weathering and Field Testing

#### 2.3.1. Outdoor Exposure Testing Results

**Figure 3:**
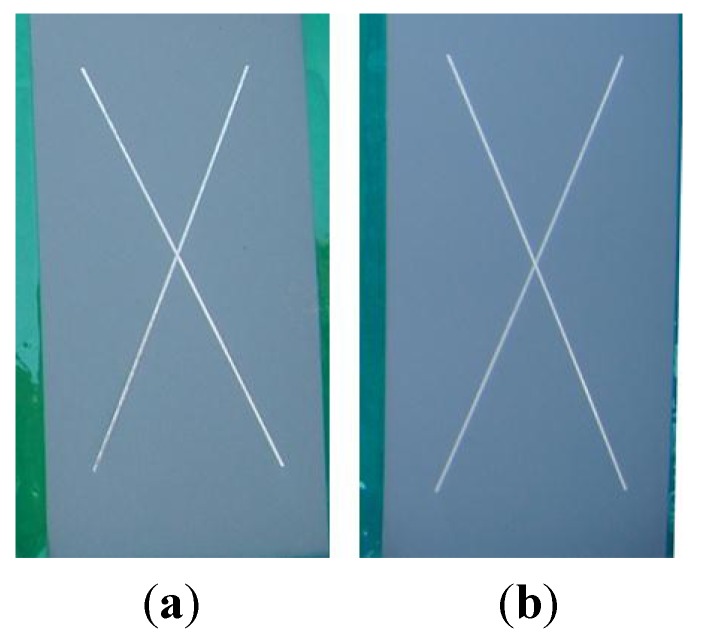
Photos after 12 months of outdoor exposure testing. (**a**) control; (**b**) BAM-PPV.

**Figure 4:**
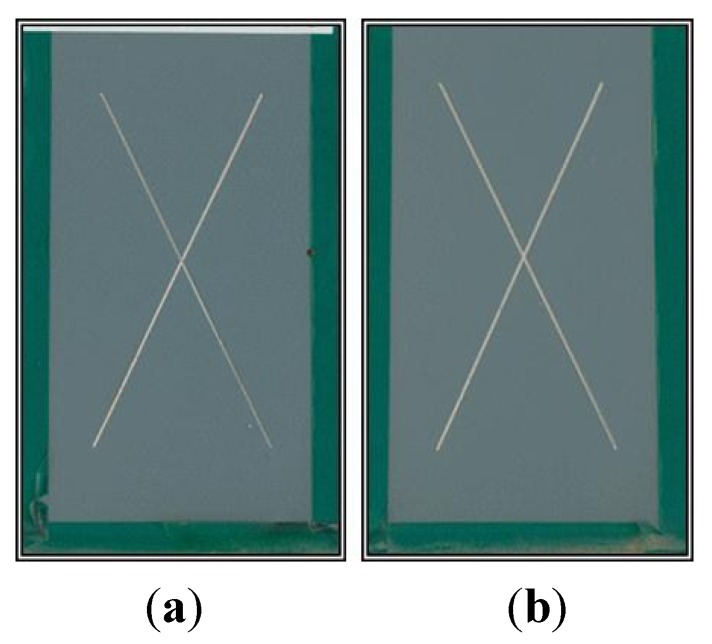
Photos after 24 months of outdoor exposure testing. (**a**) control; (**b**) BAM-PPV.

The results obtained from accelerated weathering tests conducted under controlled laboratory conditions can only give an estimate as to the corrosion resistance, degradation and adhesion characteristics of an aerospace coating. BAM-PPV aerospace coatings were subjected to outdoor weathering to determine the real effect natural weather patterns will have on aerospace coatings of this type [29]. These data, along with accelerated corrosion and weathering data, provide more detail as to the anticipated performance of the BAM-PPV coating prior to field-testing. The control and BAM-PPV samples were scribed with an X-scribe (full size) prior to outdoor exposure. There were three replicates per coating system and the results are found in Table 1. The rating system was the same as that used in NSS exposure testing, discussed above [27]. There was no corrosion, undercutting or blistering observed on the control or the BAM-PPV coated samples after 24 months of outdoor exposure. Representative samples were photographed after 12 and 24 months of outdoor exposure, and the photos are shown in Figure 3 and Figure 4.

#### 2.3.2. Field Study Results of BAM-PPV Aerospace Coatings on Air Force C-5 Cargo Aircraft Rear Hatch Door

After successful laboratory and outdoor exposure testing of the BAM-PPV aerospace coating using measurements and tests that are based on military specifications, the BAM-PPV pretreatment coating was selected for field studies by the United States Air Force (USAF). BAM-PPV was field tested on the USAF C-5 cargo aircraft rear hatch door.

The selection criteria for choosing the USAF C-5 cargo plane rear hatch door was as follows: (a) the size of the rear-hatch door provided adequate surface area such that multiple coating systems could be tested in patches side-by-side for immediate performance comparison; (b) if a serious coating failure was noted, the door could be removed and replaced easily with minimal interference using typical repair operations; (c) the C-5 maintenance group is located at Wright-Patterson Air Force Base and is immediately accessible to AFRL Materials and Manufacturing Directorate personnel, so periodic checks and performance feedback were readily available; (d) the flight performance of the system could be ascertained from the test bed which is immediately comparable to many other aircraft performance requirements and (e) BAM-PPV pretreatment coating showed comparable performance to the CCC controls in several laboratory tests.

A 1 wt% solution of BAM-PPV dissolved in Oxsol 100 was used to coat the C-5 aircraft rear hatch door. High volume low pressure (HVLP) spraying was used as the delivery system to apply the BAM-PPV solution onto the cargo door using multiple passes to get a thickness of approximately 2 μm. This coating thickness matched that used in the NSS exposure testing of BAM-PPV, discussed above. The BAM-PPV pretreatment coating was covered with the same primer and topcoat that were used in the laboratory studies. The same Cr(VI) aerospace coating was used as the control for this field demonstration. The rear hatch door was divided into four quadrants. Two quadrants were coated with BAM-PPV and the remaining two were coated with the control.

The coated door was flown for 12 months and was visually inspected every three months. The aircraft was flown under natural conditions in the Midwest and Northeast regions of the United States with one overseas flight to Europe, giving a total of 296.7 flight hours. The conditions included down-time at military depots for routine maintenance and inspection. The C-5 cargo plane was exposed to normal weather conditions found in these regions of the United States, which included rain, sleet, snow, northern coastal moisture and sun as well as wide temperature fluctuations. The door with the BAM-PPV pretreatments and controls survived the field demonstration intact without loss of adhesion or corrosion. There was no significant change in dry film thickness observed during the 296.7 flight hours. The overall assessment of this BAM-PPV coating via visual inspection over a 12 month period showed no corrosion damage to the coating, delamination, erosion effects, blistering, chalking or cracking (see Table 5). The small changes that were observed were due to dirt build-up on the coatings.

**Table 5:** Assessment of BAM-PPV field test on air force C-5 cargo plane rear hatch door.

Visual inspection of coating for:	Performance at 3 months	Performance at 6 months	Performance at 9 months	Performance at 12 months
Blistering	No blistering of coating	No blistering of coating	No blistering of coating	No blistering of coating
Delamination	No delamination of coating	No delamination of coating	No delamination of coating	No delamination of coating
Corrosion	No corrosion evident	No corrosion evident	No corrosion evident	No corrosion evident
Cracking	No cracking of exterior coating	No cracking of exterior coating	No cracking of exterior coating	No cracking of exterior coating
Color Appearance	No color change	No color change	slight color change dirt build-up	slight color change dirt build-up

## 3. Experimental Section

### 3.1. Materials

The monomer 2,5-bis(chloromethyl)-4-(hexamethylamino)-phenyl)hexamethylamine dihydrochloride and the polymer, BAM-PPV were prepared according to previously reported literature procedures [22,23,24,31,32]. 1-Chloro-4-(trifluomethyl) benzene (Oxsol 100), acetone, methanol, and Alconox were purchased from Aldrich Chemical Company (St. Louis, MO, USA) and used as received. Aluminum alloy (AA2024-T3) bare and CCC coupons were purchased from Q-Lab Corporation (Westlake, OH, USA). BAM-PPV was coated on AA2024-T3 as described below.

### 3.2. Sample Preparation

#### 3.2.1. Aluminum Substrate Preparation

Aluminum alloy (AA) (AA2024-T3) coupons (76 cm × 152 cm × 0.8 mm) were cleaned prior to coating with the BAM-PPV solution. The cleaning process consisted of washing the AA coupons with a 5 wt% Alconox solution in deionized water (DI water), followed by rinsing with DI water, methanol and a final rinse with acetone. The AA2024-T3 substrates were allowed to dry under ambient conditions.

#### 3.2.2. BAM-PPV Solution Preparation

The BAM-PPV polymer was ground to a fine powder using a commercial coffee grinder. The powder was dissolved in Oxsol 100 by stirring on a stir plate set to 300 rpm with heating (100–150 °C). After 60 min, a ~1 wt% solution was obtained. The BAM-PPV solution was filtered through coarse filter paper to remove trace amounts of insoluble impurities.

#### 3.2.3. HVLP Application of Pretreatment, Primer and Topcoats Applied on Aluminum Substrates and C-5 Hatch

The pretreatment layers were applied using a DeVilbiss GTi HVLP gun. A 1 wt% BAM-PPV solution in Oxsol 100 was used for coating all aluminum substrates. The parameters of the HVLP system were: Line Pressure 15 psi, Fluid Setting 1.5 mL, Hose inner diameter 3/8 inch, Hose length 30 ft, HVLP gun GTI, Needle size #413, Cap size #100 and Cap psi <10. Nine cross coats were used per aluminum panel for the deposition of BAM-PPV resulting in a dry coating thickness of 2.0 μm after drying under ambient conditions. The aluminum panels used in the control samples were purchased precoated with CCC.

The aerospace primer that was used to coat the BAM-PPV pretreatment was the non-Cr(VI) epoxy primer (MIL-PRF-23377, Type I, Class N). The CCC was coated with Cr(VI) epoxy primer (MIL-PRF-23377, Type I, Class C). These primers were also applied using the HVLP application method. The coatings were mixed according to manufacturer’s instructions and applied using the same parameters listed in above. Primer coatings were applied using a single cross coat to generate an overall dry film thickness of 37.5 µm. The primer coatings were then topcoated with the Air Force polyurethane Advanced Performance Coating (APC) topcoat. This coating is identified as MIL-PRF-85285 APC topcoat. This topcoat was mixed according to manufacturer instructions and applied using the same settings, but with two cross coats to generate an overall dry film thickness of 52.5 µm.

BAM-PPV was allowed a 1-h dwell time prior to application of the non-Cr(VI) epoxy primer using HVLP. BAM-PPV coatings were set-to-touch after 30 min but samples were not coated with primer until the following work day, giving these materials about 16 h between pretreatment and primer application. Topcoat was applied 4 h after primer application. Samples were left to cure at room temperature and ambient relative humidity (approximately 24 °C and 50% RH) for 14 days prior to testing. A description of the primers and topcoat is found in Table 6. Application of both BAM-PPV and control coatings to the C-5 hatch were done using the same HVLP spray method.

**Table 6:** Air Force primers and topcoat used for laboratory and field studies.

Coating	Military Specification	Description
Primer(control)	MIL-PRF-23377, Type I, Class C	Two-component, epoxy polyamide primer containing SrCrO_4_ inhibitors
Primer	MIL-PRF-23377, Type I, Class N	Non-Cr(VI) based corrosion-inhibitors solvent-borne, high solids epoxy primer
Topcoat	MIL-PRF-85285 APC	VOC-compliant, chemically curedfluoro polyurethane topcoat

### 3.3. Adhesion Testing for Aerospace Coatings

#### 3.3.1. Crosshatch Adhesion Testing Procedure

Crosshatch adhesion testing was performed to determine the dry adhesion between the substrate, pretreatment, primer and topcoat interfaces for control (CCC, Cr(VI) epoxy primer and topcoat) and for BAM-PPV coated substrates. For this test, one applies tape to a coated substrate that has been scored through the coating in a crosshatch pattern, removes the tape and examines the sample visually to determine the amount of damage to the coating. The specifics of the procedure are published elsewhere and follow UDRI/CTIO Laboratory Procedure CLG-LP-008, Tape Test Adhesion, in accordance with ASTM D 3359 [25], Standard Test Methods for Measuring Adhesion by Tape Test [25]. The descriptions of the rating scale, as taken from ASTM D 3359 [25], are given in Table 2.

#### 3.3.2. Wet-Tape Adhesion Testing Procedure

The wet-tape adhesion test measures the inter-coat and surface adhesion of organic systems immersed in water. This test was performed on pretreatment, primed and topcoated systems for AA2024-T3 substrates. UDRI/CTIO Laboratory Procedure CLG-LP-033, Wet Tape Adhesion, conformed to Federal Test Method Standard (FTMS) 141D, Paint, Varnish, Lacquer and Related Materials: Methods of Inspection, Sampling, and Testing, Method 6301, Adhesion (Wet) Tape Test [25]. There were three replicates per coating system.

A coated test panel is immersed in DI water for 24 h at ambient temperature, after immersion the coupons are dried. Within 10 min, the coating is scribed with parallel lines and an X-cut using a razor blade. An approved adhesive tape is pressed down over the scribed area, using a rubber roller, and the tape is pulled up sharply at a 180° angle. The adhesion is determined by a visual examination of the paint remaining/removed from the scribed area, using the rating scale in ASTM D3359 Method A (Table 3).

#### 3.3.3. Pull-off Adhesion Pneumatic Adhesion Tensile Test Instrument (PATTI) Procedure

For this test, a tensile stress is applied to a coating and the force is increased until the coating separates from the substrate. The test quantitatively measures the strength of the adhesion, and allows one to determine the failure mode of the coating. Descriptions of the possible failure modes are shown in Table 7. The pull-off tests were performed using a PATTI-110 system (SEMicro), and full details of the procedure, which followed UDRI/CTIO Laboratory Procedure CLG-LP-046, Tensile Adhesion, in accordance with ASTM D 4541 [26], Standard Test Method for Pull-Off Strength of Coatings Using Portable Adhesion Testers, are published elsewhere [26].

**Table 7:** PATTI coating failure descriptions.

Notation	Description	Failure Mode
T/T	Topcoat on pull stub and panel surface	Topcoat-Topcoat (Cohesion)
T/P	Topcoat on pull stub and primer on panel surface	Topcoat-Primer (Adhesion)
P/P	Primer on pull stub and on panel surface	Primer-Primer (Cohesion)
P/S	Primer on pull stub and no visible coating on panel surface	Primer-Substrate (Adhesion)
T/E	Topcoat on panel and epoxy either on panel or on stub	Topcoat-Epoxy (Adhesion)
P/E	Primer on panel and epoxy either on panel or on stub	Primer-Epoxy (Adhesion)
P/Pretreat	Primer on the stub and pretreatment on the panel	Primer-Pretreatment (Adhesion)
Pretreat/Pretreat	Pretreatment on the stub and on the panel	Pretreatment-Pretreatment (Cohesion)

### 3.4. Accelerated Weathering Testing Procedures

#### 3.4.1. Neutral Salt Spray (NSS) Testing Procedure

Coated samples were scored and exposed to NSS in accordance with UDRI/CTIO Laboratory Procedure CLG-LP-019, Salt Fog Corrosion, and ASTM B 117 [27], Standard Practice for Operating Salt Spray (Fog) Apparatus [27]. Pretreatment coated coupons were primed and topcoated and scribed with an X-scribe (full size). The coupons were exposed for 2000 h NSS, and checked for blistering, loss of adhesion, undercutting, pitting and corrosion build-up in the scribe every 500 h. The ratings system for the analysis of the samples is given in Table 4.

#### 3.4.2. Xenon arc Accelerated Weathering Test Procedure

Xenon arc weathering, performed in accordance with UDRI/CTIO Laboratory Procedures CLG-LP-036, Xenon Arc Accelerated Weathering Test, CLG-LP-019 and CLG-LP-008, was used to predict the amount of color change one would expect for a real world sample coated with BAM-PPV. The procedures used conform to ASTM G 155 [28], Standard Practice for Operating Xenon Arc Light Apparatus for Exposure of Non-Metallic Materials [28]. 

A color spectrophotometer MacBeth 7000A is used to measure the CIELAB color values for test specimens. The spectrometer settings are: (1) spectral gloss included; (2) D-65 illuminant; and (3) 10° observer. After the spectrometer is warmed up, it is calibrated using a white tile reflectance standard, a measurement is made with a color standard tile for gage, reliability, and reproducibility. Four color measurements are taken on each panel in an area free from defects, with the panel being rotated 90° between measurements. The average value for the four measurements is reported as L*, a*, and b* values in color space. These values are interpreted by ASTM D 2244 [30].

#### 3.4.3. Outdoor Exposure Testing

The ASTM D 1014 [29], Standard Practice for Conducting Exterior Exposure Tests of Paints and Coatings on Metal Substrates, was used to evaluate the BAM-PPV coating performance in outdoor environments [29]. Samples were coated, scored and placed outdoors at Wright-Patterson Air Force Base in Dayton, OH for 24 months of exposure (start date February 2007–end date February 2009). The samples were visually inspected periodically (every 3 months) for the appearance of corrosion and/or coating delamination.

## 4. Conclusions

The following conclusions can be made regarding BAM-PPV performance in both laboratory and field testing as compared to the Cr(VI) controls:

(a) The BAM-PPV has excellent adhesion characteristics in aerospace coating systems (pretreatment, non-Cr(VI) primer and APC topcoat);
(b) The BAM-PPV coating systems showed acceptable pull-off adhesion results as compared to the Cr(VI) control;
(c) The results using BAM-PPV as pretreatment coating with MIL-PRF-23377, Type I, Class N and MIL-PRF-85285 APC showed acceptable corrosion protection as compared to the Cr(VI) control under NSS exposure testing;
(d) BAM-PPV as a pretreatment coating with MIL-PRF-23377, Type I, Class N and MIL-PRF-85285 APC showed excellent corrosion protection as compared to the Cr(VI) control in outdoor exposure testing;
(e) Xenon arc testing (500 h) of BAM-PPV coating systems showed little change in color, with Δ*E* values of well less than 1;
(f) The results from field testing at Wright-Patterson Air Force Base confirmed that BAM-PPV pretreatment incorporated into a non-Cr(VI) aerospace coating on AA2024-T3 alloys performs as well as a Cr(VI) aerospace coating in real world environments.
